# Hemisphere‐specific effects of prefrontal theta‐burst stimulation on visual recognition memory accuracy and awareness

**DOI:** 10.1002/brb3.1228

**Published:** 2019-03-14

**Authors:** Ivan Carbajal, Jonathan T. O’Neil, Robert T. Palumbo, Joel L. Voss, Anthony J. Ryals

**Affiliations:** ^1^ Department of Psychology University of North Texas Denton Texas; ^2^ Department of Medical Social Sciences Northwestern University Feinberg School of Medicine Chicago Illinois

**Keywords:** brain stimulation, feeling‐of‐knowing, memory accuracy, metamemory, TMS, visual recognition

## Abstract

**Background:**

The prefrontal cortex has been implicated in episodic memory and the awareness of memory. Few studies have probed the nature and necessity of its role via brain stimulation. There are uncertainties regarding whether the hemisphere of stimulation predicts effects on memory and whether effects of stimulation are format‐specific, with most previous studies utilizing verbal/semantic stimuli.

**Objective:**

Our primary objective was to determine if theta‐burst transcranial magnetic stimulation (TBS) to prefrontal cortex modulates visual memory accuracy, visual memory awareness, or both, and whether these effects depend on brain hemisphere.

**Methods:**

We administered TBS to 12 individuals in either left prefrontal, right prefrontal, or a sham location on three separate days. We then administered a visual associative‐memory task incorporating global‐level awareness judgments and feeling‐of‐knowing (FOK) judgments on test trials for which retrieval failed.

**Results:**

Overall memory accuracy significantly improved after right hemisphere TBS compared to sham. Simultaneously, subjects were relatively underconfident after right TBS, suggesting minimal awareness of memory accuracy improvements. The correspondence between FOKs and later recognition accuracy suggested a pattern of disruption in prospective memory monitoring accuracy after left TBS.

**Conclusions:**

Our findings provide unique evidence for improved visual memory accuracy after right prefrontal TBS. These results also suggest right prefrontal lateralization for visual memory and left‐hemisphere specialization for item‐level prospective memory awareness judgments. Taken together, these results provided continued support for noninvasive stimulation to prefrontal cortex as a means of potentially improving memory and causally influencing prospective memory awareness.

## 1 INTRODUCTION

The prefrontal cortex has been associated with a variety of roles in episodic memory (e.g., Blumenfeld & Ranganath, 2007; Fletcher, Shallice, Frith, Frackowiac, & Dolan, 1998; Henson, Shallice, & Dolan, 1999; Simons & Spiers, 2003; Ranganath, Johnson, & D'Esposito, 2000; Ranganath & Gregor, 2003; Tulving, Kapur, Craik, Moscovitch, & Houle, 1994), including encoding and retrieval operations crucial for memory accuracy as well as introspective functions related to the conscious experience of memory content (e.g., Baird, Smallwood, Gorgolewski, & Margulies, 2013; Gagnon, Schneider, Grondin, & Blanchet, 2011; Fleming & Dolan, 2012; Fleming & Frith, 2014; Metcalfe & Schwartz, 2016; Modirrousta & Fellows, 2008; Wheeler, Stuss, & Tulving, 1997). Despite an enormous relevant fMRI literature, very few studies have used noninvasive brain stimulation methods to test the nature of prefrontal contributions to episodic memory. These studies have generally applied transcranial magnetic or direct current stimulation unilaterally to either left or right prefrontal cortex and identified changes in memory accuracy and/or awareness (Blumenfeld, Lee, & D'Esposito, 2014; Chua & Ahmed, 2016; Demeter, Mirdamadi, Meehan, & Taylor, 2016; Javadi & Walsh, 2012; Köhler, Buckner, & Milner, 2004). The goal of the present study was to use a within‐subjects design to compare the effects of left versus right theta‐burst transcranial magnetic stimulation (TBS) on visual episodic memory accuracy and introspective awareness in healthy adults.

Few previous studies have investigated the impact of prefrontal TBS on episodic memory (reviewed in Chua, Pergolizzi, & Weintraub, 2014; Demeter, 2016). For instance, Köhler et al. (2004) reported increased recognition hits after TBS of left but not right inferior prefrontal cortex during encoding. However, Blumenfeld et al. (2014) found increased recognition accuracy following TBS of left dorsolateral PFC but decreased recognition accuracy after TBS of left ventrolateral prefrontal cortex. We previously found no effects of bilaterally administered TBS on recognition accuracy (Ryals, Rogers, Gross, Polnaszek, & Voss, 2015), although there were effects of stimulation on memory awareness (described next). Together, these previous studies suggest it is possible that prefrontal TBS can improve recognition accuracy, although the location and hemisphere of stimulation are important variables that seem to predict the effects of TBS on memory which have not been thoroughly explored.

Likewise, few studies have investigated the role of prefrontal TBS on awareness of episodic memory. Memory awareness is the ability to introspect about the content and/or accuracy of memory processing, including a set of abilities frequently termed “metamemory” (Dunlosky & Tauber, 2016; Flavell, 1979; Metcalfe & Schwartz, 2016; Nelson, 1990). We previously found that TBS caused increases in the correspondence between judgments of memory formation success (i.e., judgments of learning,) (Koriat, 1997; Schwartz, 1994) and subsequent memory performance. This increase in the correspondence between memory awareness and memory accuracy was greater after bilateral frontopolar TBS (to Brodmann area 10) than after bilateral dorsolateral prefrontal TBS (to Brodmann area 46). In a related study using a general‐knowledge recognition memory task Chua and Ahmed (2016) recently found that prefrontal transcranial direct current stimulation improved the accuracy of feeling‐of‐knowing (FOK) responses, which are judgments made after retrieval errors that estimate the likelihood of correctly recognizing missed items on a later recognition test (Hart, 1965; Koriat, 2000; Metcalfe & Schwartz, 2016; Schwartz, Bacon, & Pillot, 2014). In both Ryals et al. (2015) and Chua and Ahmed (2016), memory awareness was modulated independently from memory accuracy, which is similar to some findings suggesting that anterior prefrontal lesions impair memory awareness without affecting memory accuracy (Chua et al., 2014; Pannu & Kaszniak, 2005). Prospective memory monitoring is particularly sensitive to prefrontal lesions (Burgess, Gonen‐Yaacovi, & Volle, 2011; Dreher, Koechlin, Tierney, & Grafman, 2008; Shimamura, 1995), including FOK responses (Metcalfe & Schwartz, 2016; Modirrousta & Fellows, 2008). Given the scarcity of previous studies, little is known regarding whether factors such as TBS location determine whether memory awareness will be affected by stimulation.

In this study we tested the effects of TBS administered to right versus left prefrontal cortex on memory accuracy and memory awareness. Memory accuracy was measured as accuracy in a recognition memory test for complex visual stimuli. Memory awareness was measured using global judgments of ability obtained immediately before testing (prospective judgments) and immediately after testing (retrospective judgments). Memory awareness was also measured using trial‐by‐trial FOK judgments. Effects of left versus right TBS were assessed relative to an active vertex control stimulation location. This location is referred to as our “sham” condition given that stimulation to this midline location is not believed to alter neural function related to memory or awareness (Jung, Bungert, Bowtell, & Jackson, 2016). Stimulation conditions were performed on three different days using a within‐subjects design. We hypothesized that TBS effects on memory accuracy and awareness would be hemisphere specific, with potential differentiation of locations that affect accuracy versus awareness, given the independence of these constructs in previous studies.

## 2 MATERIALS AND METHODS

### 2.1 Participants

Participants were recruited from the Chicago metropolitan area (*N = *15). Informed consent was obtained from all individual participants included in the study. Datasets from three participants were incomplete due to voluntary withdrawal from one or more of the stimulation conditions as a result of discomfort. This resulted in a final sample of 12 individuals (seven females; ages 21–34 years; *M* = 25.58, *SD *= 4.20). All participants were right handed, had normal or corrected‐to‐normal vision, did not report neurological or psychiatric disorders, and did not report the current use of psychoactive drugs. All participants gave written informed consent and were remunerated for their participation. All participants were eligible for MRI and transcranial magnetic stimulation (TMS) procedures based on standard MRI safety screening as well as on their answers to a TMS safety‐screening questionnaire (Rossi, Hallett, Rossini, & Pascual‐Leone, 2009).

### 2.2 MRI parameters

MRI data were collected at the Northwestern University Center for Translational Imaging, supported by the Northwestern University Department of Radiology. A Siemens 3 T TIM Trio whole‐body scanner with a 32‐channel head coil was used. Head movements were minimized with padding. A structural image was acquired to provide anatomical localization (MPRAGE T1‐weighted scans, TR = 2,400 ms, TE = 3.16 ms, voxel size = 1 mm^3^, FOV = 25.6 cm, flip angle = 8°, 176 sagittal slices). Structural MRI data were processed and stimulation targets were marked using AFNI (Cox, 1996).

### 2.3 Identification of stimulation locations and TBS parameters

**Figure 1:**
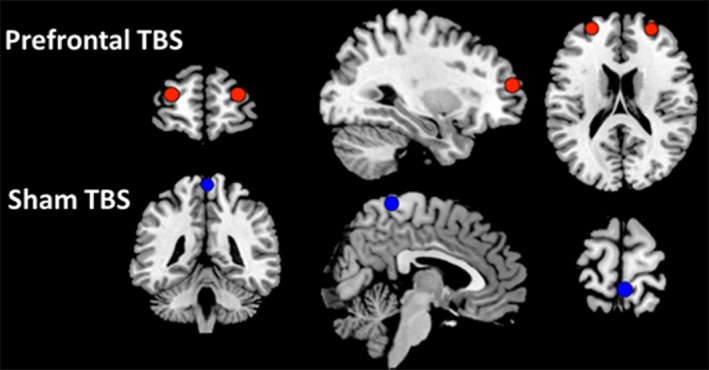
Prefrontal TBS and Sham targets. Prefrontal TBS targets corresponded to Brodmann area 9/10, (*MNI* *x* = ±30, *y* = 60, *z* = 21). Sham targets corresponded to Brodmann area 3, (*MNI* = ±4, −42, 73)

We identified three stimulation locations for each participant (Figure 1). The structural MRI for each individual was first transformed into stereotactic space using the MNI‐305 template (Evans et al., 1993). The transformation matrix was stored to enable conversion between original MRI space and stereotactic space. After transformation, targets were identified and marked for left and right prefrontal cortex (Brodmann area 9/10; *MNI* *x* = ±30, *y* = 60, *z* = 21), and for the sham location (central fissure adjacent to paracentral lobule, *MNI* = 4, −42, 73). The left and right prefrontal locations were selected after review of previous literature regarding key regions involved in recognition memory and memory awareness (Burgess, Gilbert, & Dumontheil, 2007; Cabeza et al., 2003; Chua & Ahmed, 2016; Fernandes, Moscovitch, Ziegler, & Grady, 2005; Fleming & Dolan, 2014; Gilbert, Gonen‐Yaacovi, Benoit, Volle, & Burgess, 2010; Osaka et al., 2003). Specifically, these targets straddled Brodmann areas nine and 10, and corresponded closely to a region of interest labeled “superior frontal cortex” in the Metamemory > Recognition contrast reported by Chua, Schacter, and Sperling (2009, in Table 2). Our Sham location was determined based on the approximate coordinates of the Cz (vertex) electrode from the international 10–20 EEG positioning system. Targets corresponding to stimulation locations were marked in MNI‐305 space and then transformed into original MRI space. We then overlaid the targets onto the structural MRI to provide localization during TBS (which requires a structural MRI in original space). Thus, TBS was delivered to the same MNI coordinates in every individual despite individual differences in anatomy.

TBS was delivered using a Magpro X100, MRI‐guided system with a 75‐mm figure‐of‐eight stimulation coil (Magventure, Atlanta, GA). MRI‐based anatomical targeting was achieved using frameless stereotaxy (Localite GmbH, Birlinghoven, Germany), thus allowing for high accuracy in coil positioning and real‐time monitoring of movement in order to adjust coil placement accordingly. Resting motor threshold was determined during the first session as the minimum stimulation value necessary to generate contraction of the right abductor pollicis brevis muscle for at least 60% of 10 consecutive pulses. For the treatment conditions, TBS was applied at 80% motor threshold. TBS was continuous, consisting of a series of three pulses at 50 Hz bursts separated by 160 ms (i.e., 50‐Hz triplet bursts at an ∼6‐Hz frequency). The induced current field was oriented perpendicular/anterior to the long axis of the gyrus encompassing the stimulation location.

When TBS is applied to anterior prefrontal regions, it may become uncomfortable or even painful. Therefore, prior to TBS, a test pulse was delivered and participants were allowed to discontinue stimulation if they found the stimulation aversive. During stimulation, participants were free to pull their heads away from the coil or to indicate their desire to stop the experiment at any time. Three subjects withdrew from the study based on discomfort. After indicating a willingness to proceed, TBS was administered continuously for 50 s (a total of 900 pulses) to each location. Notably, stimulation of the left and right prefrontal locations occurred for the same approximate location on each side of the head and therefore produced nearly identical subjective experiences in terms of intensity and discomfort. Therefore, any distinctions in the effects of left versus right prefrontal stimulation on performance are unlikely to have been due to differences in subjective experience.

Participants completed three experiment sessions, each of which was separated by at least 1 day (mean between‐session delay = 2.5 days, *SD *= 1.76). Different TBS targets were used in each session (left prefrontal, right prefrontal, and Sham). After stimulation, each participant completed an associative memory test using different stimuli for each of the three experimental sessions described below). The order of the stimulation locations across days was balanced across participants, with approximately the same number of participants receiving each stimulation condition for each of the three sessions.

### 2.4 Memory testing procedure

**Figure 2:**
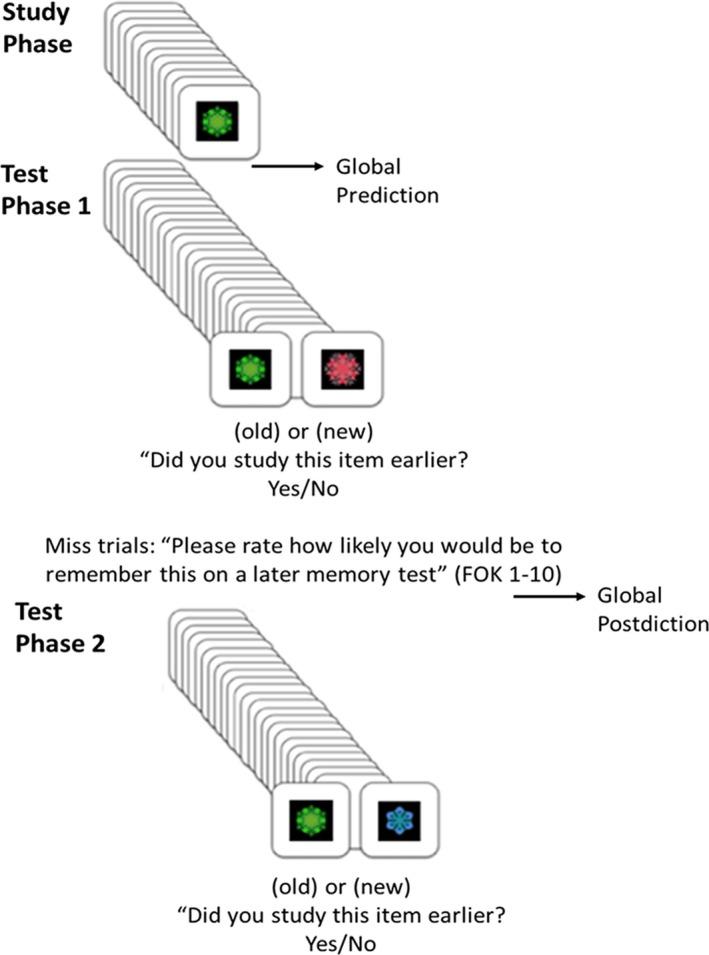
Recognition memory testing incorporating global and FOK memory awareness measures. For the study phase, participants viewed 12 unique color kaleidoscope images for 3 s each. After the study phase and before test phase 1, participants provided global predictions of prospective confidence for their upcoming test accuracy. In test phase 1 participants were shown 12 studied (old) images interleaved with 12 unstudied (new) images, and they were asked to indicate yes or no based on whether or not they studied a given image earlier. For unrecognized old images (i.e., misses), participants were given feedback about their error, and then they were asked to rate the likelihood that they would remember that item on a final recognition test on a 1–10 scale. After test phase 1, participants provided global postdictions about their retrospective accuracy confidence on the immediately preceding test. Participants then began the final recognition test containing 12 initially studied images interleaved with 12 new images and no feedback. Global predictions and postdictions were compared with actual memory accuracy for test phase 1, and FOK ratings given during test phase 1 were correlated with recognition accuracy on the final recognition test. FOK: feeling‐of‐knowing

This study used geometric color kaleidoscope images (see Voss & Paller, 2009 for a full description). Images were 13 × 13 cm and were centered on 21.60 × 27.9 cm white pages. Examples of test stimuli and an overview of the general study design are displayed in Figure 2. All tests were in bound and laminated paper notebooks, and experimenters recorded participant ratings by hand. After providing written consent, participants were comfortably seated opposite the experimenter at a distance of approximately 60 cm. Participants were read instructions detailing the procedure, and the experimenter then confirmed that they understood the instructions prior to proceeding. Stimulus order for each block was assigned using a random number generator. There was one study‐test block per stimulation condition. Each day, all procedures were completed during an approximately 2‐hr session, and there was an approximate 5‐min delay between finishing TBS and beginning the memory testing.

During the study phase of the experiment, participants viewed each item for 3 s and were instructed to remember it. Prior to testing, participants verbally provided a global prediction, an aggregate prospective estimate of their overall memory accuracy on the test to follow. During the test phase, participants completed a yes/no recognition task with 12 studied items randomly interleaved with 12 unstudied items. Participants were informed when they incorrectly answered “no” in response to a studied image (i.e., on miss trials). After feedback on each incorrect “no” response, individuals were asked to provide a 1–10 rating (using the entire scale), based on how likely they felt they would be to remember the incorrect item on a final recognition test. After the test phase, participants provided a global postdiction, an aggregate retrospective estimate of their overall memory accuracy on the test they had just taken. Participants then completed a final yes/no recognition test with 12 studied items interleaved with 12 new unstudied items and no feedback. Accuracy on this final test was used to score FOK responses made during the initial test, but was otherwise not analyzed.

## 3 RESULTS

**Figure 3:**
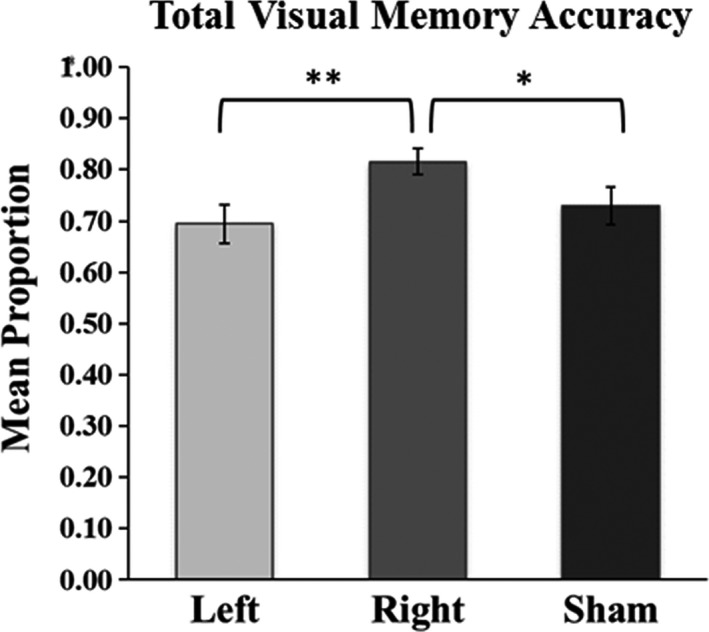
Effects of TBS on total visual memory accuracy for Test 1. Total memory was computed as the sum of hits and correct rejections divided by the total number of possible correct answers. **p < 0.01; *p < 0.05; Error bars indicate *SE*

Memory accuracy was calculated by summing the number of hits and correct rejections divided by the number of possible correct answers thus yielding a total memory accuracy score (e.g., Cave, Bost, & Cobb, 1996; Gable, Reis, & Downey, 2003; Stark & Squire, 2000; Swick, Ashley, & Turken, 2008). As displayed in Figure 3, there was a main effect of stimulation location (left prefrontal, right prefrontal, sham) on total memory accuracy scores [*F*(2, 22) = 5.39, *p* = 0.012, *p*_η_^2^ = 0.33]. Follow‐up pairwise comparisons indicated that total memory accuracy scores were significantly higher for right prefrontal compared to Sham TBS [*t*(11) = 2.29, *p* = 0.043, *d* = 0.68] compared to left prefrontal TBS [*t*(11) = 3.82, *p* = 0.003, *d* = 0.94]. Memory accuracy after Left TBS did not differ from Sham TBS (*p* = 0.45). These effects reflected a combination of total memory accuracy for recognition hits and correct rejections, although marginally significant effects were also identified for hits alone (Table 1).

**Table 1:** Mean recognition proportions for hits, correct rejections, and total memory accuracy for left, right, and sham TBS conditions

Variable	TBS condition		
	Left	Right	Sham
Hits	0.66 (0.06)	0.81 (0.15)†	0.69 (0.24)
Correct rejections	0.79 (0.11)	0.83 (0.15)	0.80 (0.15)
Total accuracy	0.69 (0.13)	0.82 (0.09)*	0.72 (0.12)

Note

Total memory accuracy was computed for each person as hits plus correct rejections divided by the total number of possible correct answers. Data are represented as mean (*SD*).

^†^ *p* = 0.06 versus Sham,

* *p* = 0.04 versus sham TBS and *p* = 0.003 versus Left TBS.

### 3.1 Global Memory Accuracy Awareness

**Figure 4:**
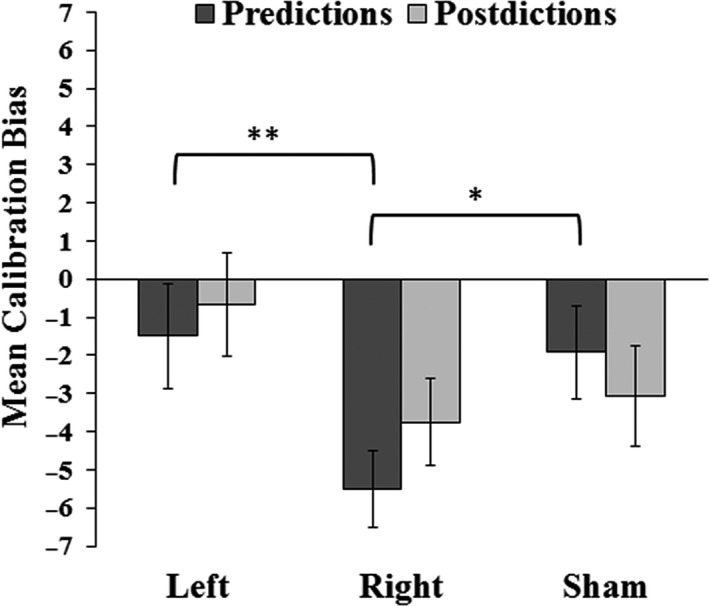
Effects of TBS on global awareness calibration bias for predictions and postdictions. Values above zero indicate global overconfidence, and values below zero indicate global underconfidence. **p < 0.01; *p < 0.05; Error bars indicate *SE*

A repeated‐measures ANOVA revealed no main effect of stimulation condition on raw mean global memory predictions [*F*(1, 11) = 0.98, *p* = 0.39] or raw mean global memory postdictions [*F*(1, 11) = 0.80, *p* = 0.46] (Table 2). This suggests that increases in memory accuracy (Figure 3) were not accompanied by corresponding increases in global memory awareness. To investigate this further, we created mean calibration scores based on the positive or negative discrepancy between both types of global judgments and accuracy out of 24 possible correct answers on Test 1. Positive calibration bias scores would indicate that subjects judged accuracy to be higher than it actually was, and vice versa for negative calibration bias scores. Prediction calibration scores, made when subjects rated how many items they thought they would get correct immediately before taking the test, varied significantly by stimulation condition [*F*(2, 22) = 4.18, *p* = 0.029, p_η_^2^ = 0.28] (Figure 4). Follow‐up pairwise comparisons indicated that prediction calibration scores were significantly more underconfident following right prefrontal TBS compared to Sham [*t*(11) = −2.38, *p* = 0.036, *d* = 1.43] and compared to left prefrontal TBS [*t*(11) = 4.23, *p* = 0.001, *d* = 2.55], with no significant difference for left prefrontal TBS versus Sham (*p* = 0.83).

**Table 2:** Mean global predictions and postdictions for visual memory accuracy

Judgment	Left	Right	Sham
Predictions	15.17 (4.88)	14.08 (4.58)	15.58 (4.50)
Postdictions	16.00 (4.69)	15.83 (5.87)	14.42 (5.68)

Note

Data are represented as mean (*SD*). Prediction values are out of 24 total possible correct answers.

In contrast, global postdiction awareness scores, made immediately after subjects completed the recognition test, did not vary significantly by stimulation condition [*F*(2, 22) = 2.63, *p* = 0.133] (Figure 4). Collectively, this indicates that in the right prefrontal TBS condition, subjects were not aware before taking the recognition test (global predictions) that accuracy was going to be higher due to stimulation, but were reasonably introspectively accurate as to accuracy levels after taking the test (global postdictions). Thus, right prefrontal TBS improved recognition accuracy without increasing subjective confidence in memory ability before taking the test and without significantly disrupting subjects’ posttest awareness of accuracy. Upon further exploration, we did discover a potential small effect of right hemisphere TBS on postdiction calibration accuracy, such that estimates were marginally different than those for left hemisphere TBS [*t*(12) = 2.17, *p* = 0.053], yet neither right nor left hemisphere postdiction calibration estimates differed statistically compared to sham (*p* = 0.13, 0.65, respectively). Thus, while it is possible that TBS had an effect on global postdiction accuracy for left hemisphere stimulation, this effect should be interpreted cautiously.

### 3.2 FOK following TBS

Feeling‐of‐knowing judgments were made on all test trials for which retrieval failed (i.e., misses), indicating the subjective confidence that missed items would be later remembered during follow‐up testing. Because memory accuracy was boosted by stimulation to right hemisphere specifically, there were few miss trials for this condition. We therefore pooled data across subjects per stimulation condition in order to calculate the relationship between FOK ratings and accuracy on follow‐up testing. For Left TBS there were a total of 45 miss trials total (an average of 3.75 misses per individual), whereas for Right TBS there were 27 miss trials total (an average of 2.25 miss trials per individual). For Sham, there were 48 miss trials total (an average of 4.0 miss trial per individual).

**Figure 5:**
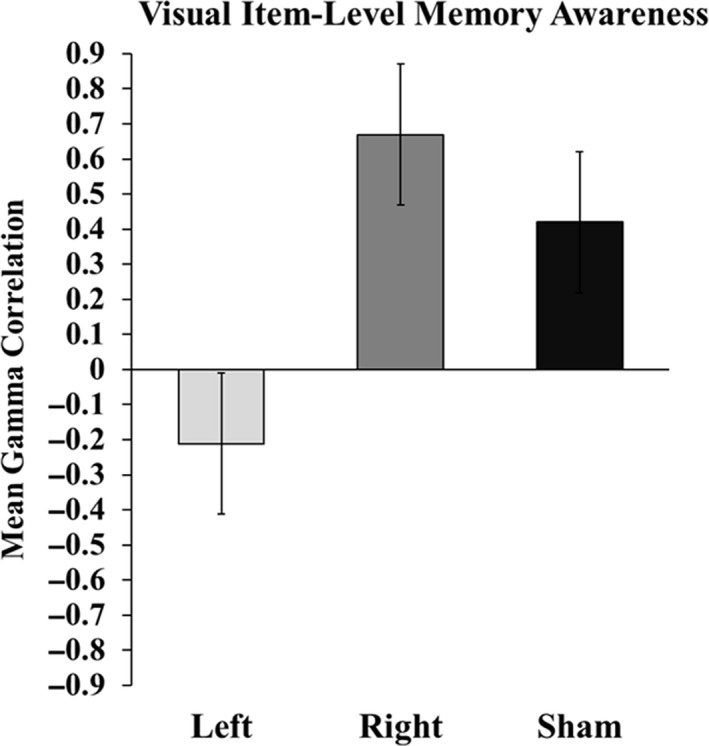
Effects of TBS on visual item‐level (FOK) memory awareness. Gamma (*γ*) values indicate the trial‐by‐trial correspondence between FOK ratings for misses during Test 1 and accuracy on the final recognition test. To compute *γ *scores, total miss trials were collapsed across individual and Test 1 FOK judgments was correlated with final recognition test accuracy. Error bars indicate the asymptotic standard error of the *γ *correlation coefficients. FOK: feeling‐of‐knowing

We computed nonparametric Goodman–Kruskal gamma (*γ*) correlations between FOK judgments and subsequent accuracy at follow‐up testing to measure resolution (Nelson, 1984). This resulted in one group‐level resolution estimate (and associated standard error of the *γ* estimate) per TBS condition (Figure 5; e.g., Dingman & Perry, 1956). Left TBS, correlations were negative (*γ *= −0.21), Right TBS, correlations were positive (*γ* = 0.67), and Sham FOK *γ* estimates were also positive (*γ* = 0.41).

Next, we computed Fisher‐transformed *Z* scores and tested for differences between our correlation estimates. Left TBS FOK accuracy was significantly lower than Sham FOK accuracy [*z* = −3.02, *p* = 0.003], and Right FOK accuracy was significantly higher than Left FOK accuracy [*z* = 4.00, *p* < 0.0001]. We did not observe a significant difference when comparing Right TBS and Sham TBS FOK accuracy, although there was a numerical difference suggesting higher accuracy for right TBS [*z* = 1.48, *p* = 0.14]. Right prefrontal TBS thus did not reliably affect FOK resolution relative to sham (both positive correlations indicating accurate resolution), whereas left prefrontal TBS was unique in reducing FOK resolution such that it was actually negative. Notably, the difference between left and right prefrontal TBS was not likely due solely to different trial counts in these conditions, as left prefrontal TBS resolution was lower than sham resolution despite relatively equivalent trial counts for these conditions.

## 4 DISCUSSION

In a recognition memory test for complex visual stimuli, right prefrontal TBS improved memory accuracy compared to sham (vertex) TBS and to left prefrontal TBS. Furthermore, global awareness judgments indicated that individuals were largely unaware before taking the test of the fact that memory accuracy was going to be higher following right prefrontal TBS. Furthermore, FOK resolution scores did not change relative to Sham for right prefrontal TBS. This stimulation condition therefore improved recognition accuracy while having no obvious effects on introspective awareness of memory accuracy. In contrast, left prefrontal TBS was associated with reduced FOK resolution compared to right prefrontal TBS and sham TBS, with no effects on memory accuracy. Prefrontal TBS effects were therefore hemisphere‐specific, with selective accuracy improvements following right prefrontal TBS and selective introspective FOK impairments following left prefrontal TBS.

Our results add to the relatively small literature on episodic memory changes due to prefrontal TBS. Interestingly, Köhler et al. (2004) reported increased recognition hit rates after left but not right inferior prefrontal TBS, which is opposite to our findings in terms of hemisphere. Likewise, Blumenfeld et al. (2014) found improved recognition accuracy following left dorsolateral prefrontal TBS. However, it is notable that Köhler et al. (2004) and Blumenfeld et al. (2014) used verbal memoranda whereas we used complex visual memoranda that did not support verbalization (i.e., complex geometrical patterns with no preexisting memory representations). Therefore, our findings support the idea that left versus right hemispheres are specialized for memory of verbal versus visuospatial information, respectively (e.g., Brewer, Zhao, Desmond, Glover, & Gabrieli, 1998; Broca, 1861; Kelley et al., 1998; Sperry, 1974), and that memory enhancements caused by prefrontal TBS are specific to the hemisphere specialized to the memoranda being used. It is possible that our recent study in which TBS was applied to both left and right hemispheres as the same condition (i.e., bilateral stimulation) identified no effects on memory accuracy because hemisphere‐appropriate stimulation was canceled out by hemisphere‐inappropriate stimulation. Indeed, here we found numerical decrease in accuracy following left prefrontal TBS, suggesting that stimulating the hemisphere not matched to memoranda could be harmful. However, another difference across studies is that Ryals et al. (2015) used item‐item associations as memoranda whereas other studies (Blumenfeld et al., 2014; Köhler et al., 2004, and the present study) used item recognition, and prefrontal TBS effects could differ for item recognition versus item‐item association recall. Future research should systematically compare stimulation hemisphere, verbal versus visual stimulus formats, and item versus associative information.

Interestingly, the present results also add to a growing body of evidence that memory accuracy and memory awareness may be at least partially independent within prefrontal cortex. Ryals et al. (2015) reported increased awareness for prospective judgments of learning after bilateral frontopolar TBS despite no effect on memory accuracy. Likewise, Chua and Ahmed (2016) reported modulation of FOK‐based memory monitoring in a semantic recognition task after left hemisphere transcranial direct current stimulation despite no changes in memory performance. Prior neuropsychological findings provide compelling evidence that anterior prefrontal lobe lesions impair memory awareness while often leaving memory performance intact (e.g., Chua et al., 2014; Pannu & Kaszniak, 2005). Additional evidence suggests that damage to anterior prefrontal cortex impairs prospective memory monitoring, such as that involved in FOK judgments, selectively (e.g., Burgess et al., 2011; Dreher et al., 2008; Metcalfe & Schwartz, 2016; Modirrousta & Fellows, 2008; Shimamura, 1995). Thus, different regions of prefrontal cortex are likely related to operations that support memory accuracy versus memory awareness, although additional research is needed to determine the degree to which these distinctions depend on factors such as memoranda, test format, etc. TBS is a useful tool to this end as it allows for the relatively selective modulation of specific regions in groups of individuals, whereas organic lesions have several obvious limitations including the diffuse nature of overlapping multiple brain regions, variability in time after lesion occurrence, heterogeneity in underlying neuroanatomy between individuals, and an inability to selectively lesion cortex in humans (e.g., Fleming & Dolan, 2014; Rorden & Karnath, 2004).

Noninvasive stimulation is particularly powerful when combined with neuroimaging techniques in order to identify changes resulting from perturbation to network regions (Opitz, Fox, Craddock, Colcombe, & Milham, 2016). For instance, not only are many prefrontal subregions implicated in cognitive monitoring, but also mounting evidence suggests that regions including anterior cingulate, insula, and posterior parietal cortex are involved in metacognitive awareness as well (Fleming & Dolan, 2012; Hu et al., 2017; Le Berre et al., 2010). Another promising avenue for future work involves determining noninvasive stimulation effects on event‐related and oscillatory electrophysiological signatures of memory monitoring and control (Müller et al., 2016; Paynter, Reder, & Kieffaber, 2009; Wokke, Cleeremans, & Ridderinkhof, 2017).

One potential limitation of our study was that we used relatively few trials in our memory testing procedure. However, a brief memory assay is also advantageous given the potential nonstationary effects of TBS on neural and cognitive processing. That is, continuous TBS protocols such as those used in this study provide modulation in cortex underlying the stimulation site that outlasts the period of stimulation for approximately 60 min (e.g., Huang, Edwards, Rounis, Bhatia, & Rothwell, 2005), but little is known regarding the time course of modulation. Brief testing helps ensure that the effects of stimulation are likely uniform across the testing interval and similar across individuals. An additional potential limitation in our study is that we pooled across individuals to create FOK estimates for our sample rather than calculating estimates for each individual and then computing a mean FOK estimate. A rather surprising yet complicating factor for this analysis was that TBS to the right hemisphere substantially improved accuracy, thus the miss trials necessary to compute individual FOKs in the traditional manner were substantially reduced. Indeed, Schwartz, Boduroglu, and Tekcan (2016) recently suggested that collecting FOKs after each test trial regardless of response accuracy may be one way to circumvent this limitation of the FOK procedure. This could be particularly useful in situations such as the current one, in which beneficial effects of TBS on memory accuracy resulted in miss trial reductions and therefore limited analyses of FOK resolution. As such, while our reported FOK effects are quite interesting with respect to hemispheric differences in awareness based on stimulation, they should be interpreted with caution and need to be replicated in subsequent studies with greater counts of FOK ratings. Finally, the current experimental design used FOK judgments combined with global judgments, and we cannot rule out some influence of feedback given on FOK (i.e., miss trials) on global postdictions. Future work should continue to explore the utility of global judgments under memory testing situations in which no feedback is given to ensure a more process‐pure measure of global postdiction accuracy.

Humans are notoriously overconfident in estimations of their own memory abilities, and overconfidence can lead to serious consequences in many aspects of life (Berner & Graber, 2008; Dunlosky & Rawson, 2012; Genon et al., 2016; Glenberg & Epstein, 1985; Kruger & Dunning, 1999). Our results offer support for TBS as one means of potentially improving episodic memory as well as modulating prospective visual memory awareness noninvasively. This has implications for efforts to improve quality of life through cognitive neurorehabilitation in a number of disorders. Importantly, the effects and potential benefits of TBS administered to prefrontal cortical regions depends on stimulus modality, the nature of memory awareness being measured, and whether or not awareness is indexed on a global level or a trial level.

## CONFLICT OF INTEREST

None declared.

## AUTHOR CONTRIBUTIONS

A.J.R. and J.L.V. conceived the study, performed the study, performed analyses, and wrote the manuscript. J.LV. Secured funding for the project. R.T.P. and J.T.O. performed the study and assisted with analyses. I.C. assisted with analyses and manuscript revision.
